# 

**Published:** 1923-07

**Authors:** 


### HOSPITAL and HEALTH

REVIEW

*Established 1886* | *No* 1864. *Vol. LXXII.*

*New Series.* Vol. II. No. 22 | *July* 1923 | Price Sixpence

CONTENTS.

| | PAGE |
|---|---|
| The Hospital's Paying Guest | 253 |
| Are We Over-Drugging Ourselves? | 254 |
| Notes and Comments | 255 |
| St. Bartholomew's Octo-Centenary | 259 |
| Panel Pay and Extended Services | 261 |
| The Years That Count. By George H. Green, B.Sc., B.Litt., Lecturer in Education, University College of Wales | 262 |
| The Public Health: Interviews with Local Authorities—Norwich | 263 |
| A Laundry Maid and a Hospital Epidemic | 265 |
| Hospital Progress and Finance | 266 |
| Extension of University College Hospital | 267 |
| The Venereal Disease Inquiry | 268 |
| Correspondence | 269 |
| Health Insurance Notes | 271 |
| Sheffield Hospital Conference | 272 |
| Heatherwood Hospital, Ascot | 282 |
| Reviews of Books | 283 |
| Echoes of the Month | 286 |
| Hospital and Health News | 287 |
| Public Health in Parliament | 288 |
| Recent Appointments | 288 |

